# Striped bodypainting protects against horseflies

**DOI:** 10.1098/rsos.181325

**Published:** 2019-01-16

**Authors:** Gábor Horváth, Ádám Pereszlényi, Susanne Åkesson, György Kriska

**Affiliations:** 1 Environmental Optics Laboratory, Department of Biological Physics, ELTE Eötvös Loránd University, 1117 Budapest, Pázmány sétány 1, Hungary; 2 Hungarian Natural History Museum, Department of Zoology, Bird Collection, 1083 Budapest, Ludovika tér 2-6, Hungary; 3 Department of Biology, Centre for Animal Movement Research, Lund University, Ecology Building, 223 62 Lund, Sweden; 4 MTA Centre for Ecological Research, Danube Research Institute, 1113 Budapest, Karolina út 29-31, Hungary; 5 Biological Institute, ELTE Eötvös Loránd University, 1117 Budapest, Pázmány sétány 1, Hungary

**Keywords:** bodypainting, horsefly, striped body patterns, zebra, visual protection, visual ecology

## Abstract

Bodypainting is widespread in African, Australian and Papua New Guinean indigenous communities. Many bodypaintings use white or bright yellow/grey/beige stripes on brown skin. Where the majority of people using bodypainting presently live, blood-sucking horseflies are abundant, and they frequently attack the naked brown regions of the human body surface with the risk of transmitting the pathogens of dangerous diseases. Since horseflies are deterred by the black and white stripes of zebras, we hypothesized that white-striped paintings on dark brown human bodies have a similar effect. In a field experiment in Hungary, we tested this hypothesis. We show that the attractiveness to horseflies of a dark brown human body model significantly decreases, if it is painted with the white stripes that are used in bodypaintings. Our brown human model was 10 times more attractive to horseflies than the white-striped brown model, and a beige model, which was used as a control, attracted two times more horseflies than the striped brown model. Thus, white-striped bodypaintings, such as those used by African and Australian people, may serve to deter horseflies, which is an advantageous byproduct of these bodypaintings that could lead to reduced irritation and disease transmission by these blood-sucking insects.

## 1. Introduction

**Figure 1.**
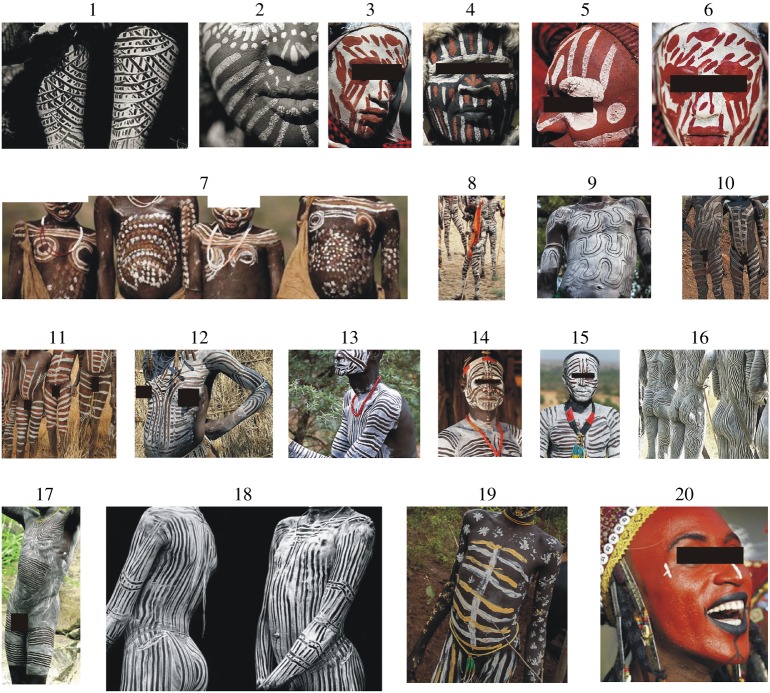
Selection of typical bodypainting patterns of different African tribes. The geographical site (continent, region with horsefly references) of these tribes and the websites these pictures were obtained from are given in table 1. These pictures were cut from larger images and personal characteristics (eyes, breasts, sex organs) have been masked by black rectangles.

**Figure 2.**
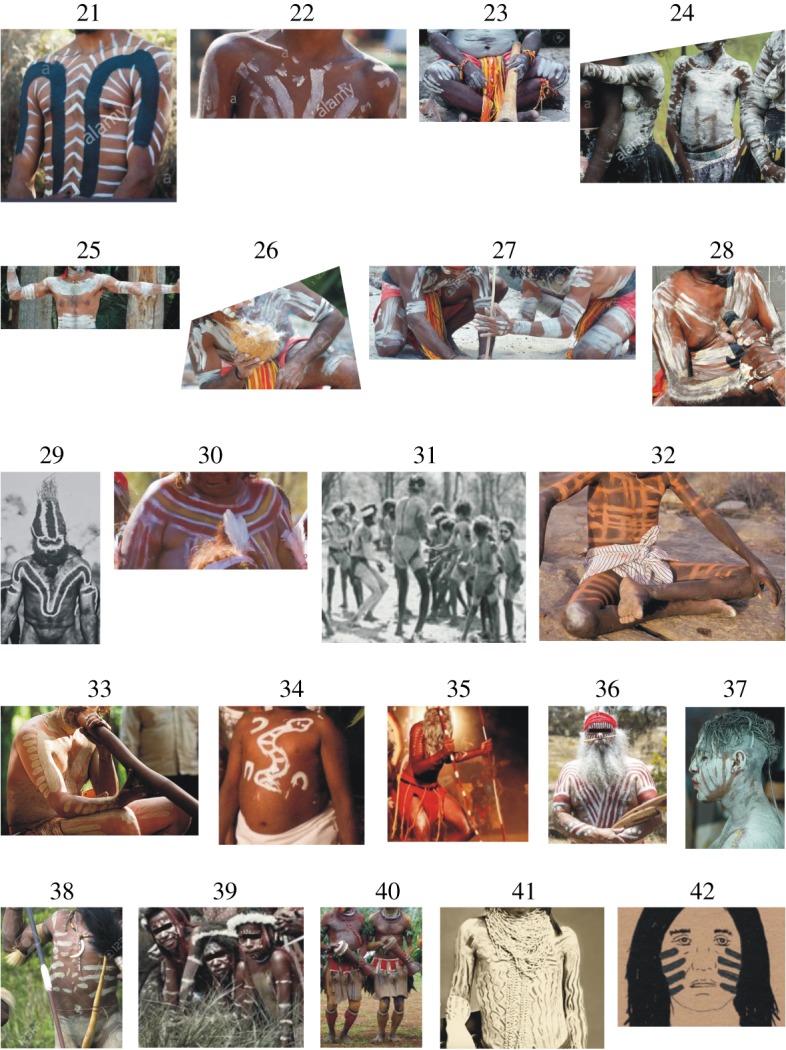
Selection of typical bodypainting patterns of different tribes living in Australia (21–37), Papua New Guinea (38–40) and North America (41–42). The geographical site (continent/island, region with horsefly references) of these tribes and the websites these pictures were obtained from are given in table 1. These pictures were cut from larger images and the eyes have been masked by black rectangles.

Members of different tribes living in Africa, Australia, Papua New Guinea and North America frequently painted their brown naked body surfaces with white or bright yellow/grey/beige stripes [1–6]. The patterns (e.g. stripy, spotty, wavy and checkered) of these paintings are extremely diverse (figures 1 and 2, table 1) and are used as body decoration, for emotional expression or as marks to signify personal identity and/or group affiliation [16,17]. The stripes of these bodypaintings are similar to the stripes occurring on the pelage of zebras and okapis. Cultural reasons may determine their designs [18,19], but it has been suggested that they could also serve a function in heat regulation or as camouflage [20,21].

**Table 1.** Geographical sites (continent/island, region with horsefly references) of the tribes which use bodypaintings and the picture numbers in figures 1 and 2 with website addresses of the original pictures.

continent/island	region (horsefly reference)	tribe	picture in figures 1 and 2 [website address]
Africa	Kenya [7]	Kikuyu	1 [1], 2 [2], 3 [3], 4 [4], 5 [5], 6 [6]
		Masai	7 [7], 8 [8]
	Ethiopia [8,9]	Mursi	9 [9], 10 [10], 11 [11]
		Omo	12 [12]
		Karo	13 [13], 14 [14], 15 [15]
		Surma (Suri)	16 [16], 17 [17], 18 [18], 19 [19]
	Sahel region, Africa [10]	Wodaabe	20 [20]
Australia [11–13]	dispersed on the whole continent	Anangu	21 [21], 22 [22], 23 [23], 24 [24], 25 [25], 26 [26], 27 [27], 28 [28]
	Central Australia	Arrernte	29 [29]
	Northern Territory	Warlpiri	30 [30]
		Yolngu	31 [31]
		Yanyuwa	32 [32]
	North Queensland Western Australia	Kuku Yalanji	33 [33]
		Jarlmadangah	34 [34]
		Mowanjum	35 [35]
	Southern-central Australia	Ngarrindjeri	36 [36]
	Southeast Australia	Wurundjeri	37 [37]
Papua New Guinea	Island [12,14]	Baruya	38 [38], 39 [39]
		Huli	40 [40]
North America	California [15]	Cocopah	41 [41]
		Kumeyaay	42 [42]

Website address of the pictures in figures 1 and 2.

[1] https://www.gettyimages.com/detail/news-photo/kikuyu-woman-in-kenya-on-july-10–2009-the-kikuyu-are-the-news-photo/120399172#/kikuyu-woman-in-kenya-on-july-10-2009-the-kikuyu-are-the-country-s-picture-id120399172.

[2] https://www.gettyimages.com/license/120399174

[3] https://www.gettyimages.com/license/120399169

[4] https://www.gettyimages.com/license/120399173

[5] https://www.gettyimages.com/license/120399171

[6] https://www.gettyimages.com/license/120399177

[7] https://hu.pinterest.com/pin/386957792959941519/

[8] https://www.pinterest.co.kr/pin/476044623084648397/

[9] https://hu.pinterest.com/pin/401453754258731590/

[10] http://yourshot.nationalgeographic.com/photos/7181740/

[11] https://www.alamy.com/stock-photo-mursi-boys-in-the-lower-omo-valley-of-ethiopia-68214304.html

[12] http://pixdaus.com/body-paint-of-omo-tribes-ethiopia-jeremy-hunter-olympus-euro/items/view/228323/

[13] https://www.gettyimages.fi/detail/photo/ethiopia-omo-valley-duss-an-elder-of-the-high-res-stock-photography/140332582

[14] https://hu.pinterest.com/pin/453948837415968602/

[15] https://hu.pinterest.com/pin/806918458211254877/

[16] https://www.pinterest.co.kr/pin/318840848590183934/?lp=true

[17] https://www.pinterest.co.kr/pin/362821313711784109/

[18] https://www.pipermackayphotography.com/2015/12/suri-portraits/suri-body-paint-4/

[19] https://www.flickr.com/photos/48286994@N05/8389998658

[20] https://www.dnbstories.com/2017/05/the-wodaabes-wife-stealing-dance.html

[21] https://furniture.digitalassetmanagement.site/edit

[22] https://www.alamy.com/stock-photo-portrait-of-a-young-aboriginal-boy-in-tribal-body-paint-laura-queensland-35476674.html

[23] https://hu.depositphotos.com/58933205/stock-photo-yugambeh-aboriginal-body-coverd-with.html

[24] https://www.alamy.com/stock-photo-aborigines-in-traditional-body-paint-wait-for-a-dance-ceremony-75508208.html

[25] https://www.123rf.com/photo_45816580_portrait-of-one-yugambeh-aboriginal-warrior-man-preform-aboriginal-culture-martial-art-during-cultur.html?fromid=US9xUTdwNWRnYVdpRTl4NUttMmpYUT09

[26] https://www.123rf.com/photo_45816537_portrait-of-one-yugambeh-aboriginal-warrior-demonstrate-fire-making-craft-during-aboriginal-culture-.html?fromid=US9xUTdwNWRnYVdpRTl4NUttMmpYUT09

[27] https://www.123rf.com/photo_45816619_group-of-yugambeh-aboriginal-warriors-men-demonstrate-fire-making-craft-during-aboriginal-culture-sh.html?fromid=US9xUTdwNWRnYVdpRTl4NUttMmpYUT09

[28] https://www.123rf.com/photo_19573458_aboriginal-man-performing-for-passing-tourists-australia-melbourne.html?fromid=US9xUTdwNWRnYVdpRTl4NUttMmpYUT09

[29] http://spencerandgillen.net/objects/50ce72e9023fd7358c8a8534

[30] https://blog.parksaustralia.gov.au/2012/07/19/southern-tanami-becomes-indigenous-protected-area/southern-tanami-indigenous-protected-area-launchsangster%C2%92s-bore-10-july-2012/

[31] http://sydney.edu.au/news/84.html?newsstoryid=4238

[32] https://ozoutback.com.au/Australia/marndiwala/slides/1992122310.html

[33] https://www.thalabeach.com.au/kuku-yalanji/

[34] http://www.abc.net.au/news/2014-09-26/jarlmadangah-dancers-men-281-of-129.jpg/5773034

[35] http://desertriversea.com.au/art-centres/mowanjum-aboriginal-art-and-culture-centre

[36] https://www.ngarrindjeri-culture.org/major-sumner/

[37] http://www.abc.net.au/news/2013-04-25/indigenous-performer-opens-anzac-ceremony-in-melbourne/4652014

[38] https://www.123rf.com/photo_11201177_new-guinea-indonesia-december-28-unidentified-warriors-of-a-papuan-tribe-in-traditional-clothes-and-.html?fromid=bWV0WG8wT2tocDUrMUQrZ3JTUWNaZz09

[39] https://www.franceculture.fr/emissions/tout-un-monde/les-baruya-du-neolithique-la-modernite

[40] http://www.new-guinea-tribal-art.com/wp/index.php/2012/01/29/huli-tribe/

[41] http://www.kumeyaay.info/body_paint_pigments/indian_body_paint.html

[42] http://www.kumeyaay.info/body_paint_pigments/index.html

In areas where people using bodypaintings presently live, there are usually abundant horsefly populations (table 1, [7–14,22]), and because the main part of the body surface is usually exposed when wearing these paintings, they are vulnerable to attack by blood-sucking horseflies (Tabanidae) as well as other insect parasites, like tsetse flies (Glossinidae) and black flies (Simuliidae). Horseflies prefer to attack and suck the blood of homogeneous dark-coated mammals [23]. The darker the host animal, the more attractive it is to horseflies. However, the attractiveness to horseflies decreases with increasing heterogeneity of the body pattern, such that host animals with thinner and more numerous stripes on their coats attract fewer horseflies than hosts with wider and fewer stripes [24,25]. The most striking striped mammals are zebras, in which it has been shown that their striped body pattern reduces their attractiveness to horseflies as compared to both homogeneous dark and white/bright host animals, the latter being much less attractive to these parasites than the former [26]. Animals with spotted coats are similarly less attractive to horseflies than animals with less spotty or homogeneous fur patterns [27].

Owing to the resemblance between zebra stripes and the bright stripes of traditional bodypainting, we hypothesized that (A) these bodypaintings have the advantageous effect that they protect humans visually against the attack of blood-sucking horseflies. In a field experiment performed in Hungary, we tested hypothesis A with the use of human models with different colours/patterns and postures.

It has previously been found that polarization of the reflected light is a significant factor in the attractiveness of host animals and water to horseflies: light with a high degree of polarization and arbitrary direction of polarization attracts only host-blood-seeking females, while horizontally polarized light attracts water-seeking males and females [28–34]. In our field experiment, we studied the attractiveness of standing or lying human models to horseflies. The elongated semi-cylindrical shape of the human body differs considerably from the shape of the natural host animals of horseflies (e.g. horses and cattle). (B) Whether the standing (vertical) or lying (horizontal) posture of humans does influence its attractiveness to horseflies. The reflection–polarization characteristics of these two quite different postures may be considerably different. (C) We hypothesized that homogeneous dark human bodies in a standing posture reflect mainly vertically polarized light, and thus may attract only blood-seeking female horseflies. (D) On the other hand, homogeneous dark human bodies in a lying posture may reflect predominantly horizontally polarized light which imitates the reflection–polarization characteristics of water surfaces, and therefore may attract both male and female water-seeking horseflies. To test hypotheses B–D, we measured and compared the reflection–polarization patterns and the attractiveness of different human models to horseflies.

Bodypaintings were traditionally made from readily available natural ingredients, such as clay, chalk, minerals, ash, oil (from animals and/or plants), cattle dung, urine and plants [5]. Nowadays, some of these natural components are replaced by artificial industrial commercial paints and oils. The bodypainting is smoothed on the skin with fingers, sticks or grass [17]. Although several of these ingredients result in different colours, the most frequently used colours of bodypaintings are white or bright yellow and brown or grey due to the major contribution of chalk, clay, ash and various bright minerals. White is often ritually applied to both boys and girls to initiate them into society [16]. For this reason, we used white stripes on a dark brown human model in our field experiment.

## 2. Material and methods

### 2.1. Multiple-choice experiment

**Figure 3.**
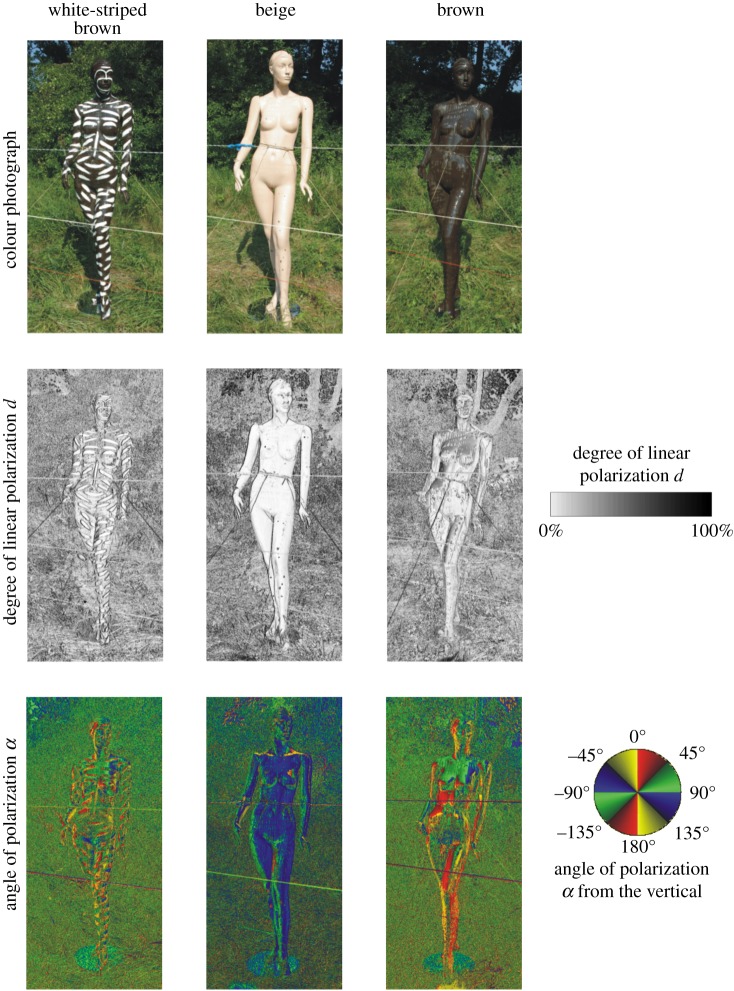
Reflection–polarization characteristics of standing human models. Colour photographs and patterns of the degree of linear polarization *d* and the angle of polarization *α* (clockwise from the vertical) of the sunlit sticky white-striped brown (*a*), beige (*b*) and brown (*c*) human models in standing posture used in the choice experiment measured by imaging polarimetry in the blue (450 nm) part of the spectrum. The optical axis of the polarimeter was horizontal.

Our field experiment was performed between 22nd June and 16th August 2015 in a meadow near Szokolya (47° 52′ N, 19° 00′ E) in Hungary, where there are numerous horsefly species in the summer [26,27,29,32]. Three different plastic human models were used: homogeneous dark brown, dark brown with white stripes and homogeneous light beige (figure 3). The light beige model was used as a control and was intended to model people with fair skin, whereas the dark brown models were intended to model people with darker skin such as members of the indigenous tribal communities of Africa, Australia and North America. All models had an identical and realistic shape and size (height = 180 cm, width = 50 cm, thickness = 25 cm). The models were produced by the Figura Dekor Ltd. (Budapest, Hungary). The white stripes (width = 4 cm, separation = 4–5 cm, length = 10–50 cm) produced with a common oil paint (Trikolor white, Trilak-Haering Ltd., Budapest) on one of the two brown models mimicked the stripes of African and Australian tribal bodypaintings (figures 1 and 2).

From 22nd June to 19th July 2015 the human models were set up in the meadow in a standing posture 10 m from a group of trees and bushes (electronic supplementary material, figure S1A). Between 20th July and 2nd August 2015, the models were placed in a lying posture on the ground with their stomach down (electronic supplementary material, figure S1B), while from 3rd to 16th August 2015 they were placed in a lying posture on their backs (electronic supplementary material, figure S1C). It is well known that water-seeking female and male horseflies are attracted to horizontally polarizing surfaces [35]. Our human models in their lying posture reflected also horizontally polarized light, and thus they mimicked this situation. On the other hand, female horseflies looking for host animals to suck blood from are preferentially attracted to dark objects [35]. Our human models in their standing posture imitated this case. This was the reason we used models in both postures. In cloudless weather, the models were lit by direct sunlight from sunrise (approx. 5.30 h = Universal Time Coordinated + 2 h = local summer time) until approximately 11.30 as well as from nearly 14.30 to sunset (approx. 20.30), while they were in the shade of the neighbouring vegetation between approximately 11.30 and 14.30.

The models were covered with a thin, transparent and colourless adhesive (mouse-trapping glue, BabolnaBio^®^ mouse trap), which was refreshed weekly. This glue is weather-proof and contains no diluent; its continuous stickiness is ensured by special macromolecules, which cannot evaporate. Thus, it can be considered odourless for animals and humans. Even if this glue was slightly attractive to horseflies, this would not influence the results of our field experiment as all three human models were covered with the same adhesive.

Every two days, we collected horseflies from the numerous different insects (mainly flies) trapped by our sticky human models (electronic supplementary material, figure S1D–F; figure S1G–I) and all the other remaining insect carcasses were removed from the test surfaces. Since practically only the brown regions of the test surfaces attracted and trapped horseflies, their carcasses were almost indiscernible from the brown background. Thus, the possible attraction of other living horseflies by the carcasses of trapped ones was minimal. In our earlier choice experiments with horseflies performed in the field, this method was also used effectively [23,26,27,36].

We determined the sex of trapped horseflies on the basis of the existence (female) or non-existence (male) of an ommatidia-free thin zone between the two compound eyes. Although the specific species of trapped horseflies could not be identified because of the damages to their body during collection from the insect-monitoring glue, they were horseflies (Tabanidae). Based on the taxonomical results of our other field experiments in which horseflies were trapped and monitored in the same site [33,34], it is likely that the horseflies trapped in our present field experiment belonged to the following species: *Atylotus loewianus* Villeneuve 1920, *Tabanus tergestinus* Egger 1859, *Tabanus bovinus* Linnaeus 1758, *Tabanus maculicornis* Zetterstedt 1842, *Tabanus bromius* Linnaeus 1758, *Haematopota pluvialis* Linnaeus 1758. When the trapped horseflies were collected every two days from the sticky human models, we also removed all other non-horsefly insects, the body size of which was not smaller than 1 mm, in order to keep the body surface of the models as clean as possible. To eliminate a possible site effect, the places of the models were randomized after each horsefly collection.

After being trapped by the insect-monitoring glue, some horsefly carcasses slipped off the human models due to the decreased viscosity of the glue that had been warmed by the sun. Therefore, we also collected these horseflies from the ground below the models, and included these horseflies in the count.

### 2.2. Imaging polarimetry

Horseflies are polarotactic insects that are attracted to (among other cues) linearly polarized light when looking for water (by horizontal polarization) and host animals (by darkness and degree of polarization) [35]. Therefore, we were interested in the reflection–polarization characteristics of the human models used in our field experiment. The reflection–polarization patterns of the human models were measured with imaging polarimetry in the red (650 nm), green (550 nm) and blue (450 nm) parts of the spectrum. This method has been described in [37,38].

### 2.3. Statistical analysis

We compared the numbers of horseflies captured by our three sticky human models using factorial ANOVA with Tukey HSD post hoc tests implemented in Statistica 7.0. In the statistical analysis, the independent variables were the posture (standing or lying) and the colour (white-striped brown, beige, brown) of the human models as well as the sex of the horseflies.

## 3. Results

Figures 1 and 2 show a selection of typical bodypainting patterns of different tribes living in Africa (figure 1), Australia (figure 2/21–37), Papua New Guinea (figure 2/38–40) and North America (figure 2/41–42). The geographical sites (continent/island, region with horsefly references) of these tribes and the website addresses of the original pictures are given in table 1. According to our survey, almost all bodypaintings contain bright (e.g. white, yellow, orange, grey) stripes of different lengths and widths. Hence, we used a white-striped brown human model in our field experiment. It is clear from table 1 that horseflies occur in the regions where the tribes using bodypaintings live.

**Figure 4.**
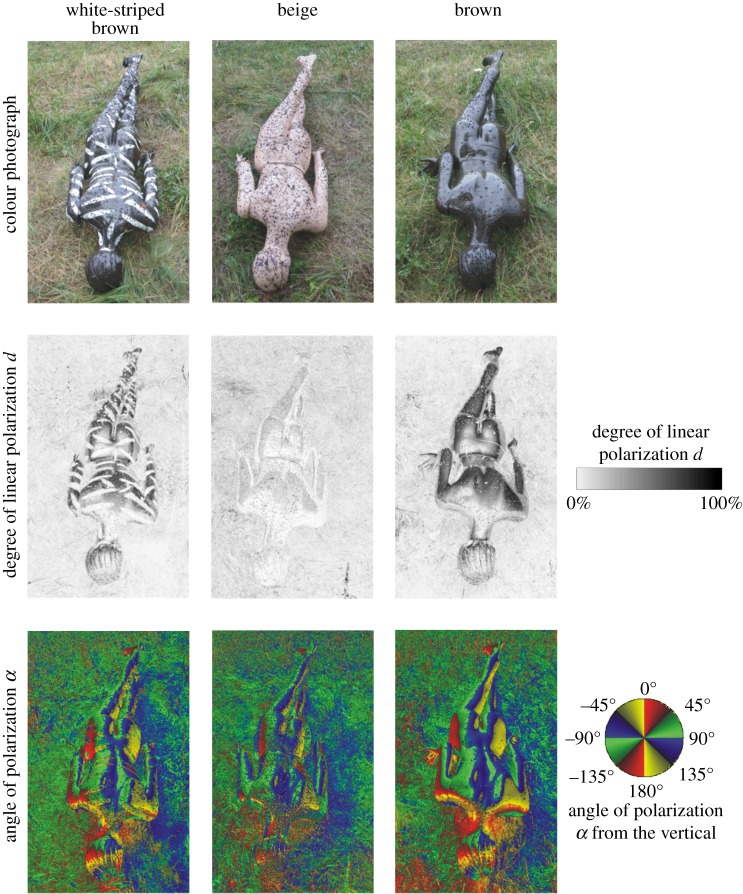
Reflection-polarization characteristics of lying human models. As figure 3 for the shady sticky human models lying on the ground with stomach down, when the optical axis of the polarimeter was −35° from the horizontal and parallel to the long axis of the models.

According to our imaging polarimetric measurements (figures 3 and 4 and electronic supplementary material, figures S2 and S3), the brown human model and the brown regions of the white-striped brown model reflected light with the highest degrees of polarization (*d* < 90%). The beige model reflected weakly polarized light with *d* < 20%, and the white stripes were practically unpolarizing (*d* < 5%). The *d* of model-reflected light was highest when the light was reflected from the Brewster's angle *θ*_B_ = arc tan (*n*) ≈ 56.3° from the local normal vector of the curving body surface, where *n* ≈ 1.5 is the refractive index of plastic, of which the models were composed. The *d* of light reflected from a given model was highest in the blue (450 nm) and lowest in the red (650 nm) spectral range. The direction (or angle) of polarization of model-reflected light changed continuously along the continuously curving body surface of the models in such a way that it was always perpendicular to the local plane of reflection determined by the observer (polarimeter, polarization-sensitive horsefly), the point observed and the incident light beam with the largest intensity (sunlight, skylight and leaflight). Contrary to the patterns of the degree of polarization, the patterns of the angle of polarization were robust, depending only slightly on the wavelength of light (figures 3 and 4 and electronic supplementary figures S2 and S3).

**Figure 5.**
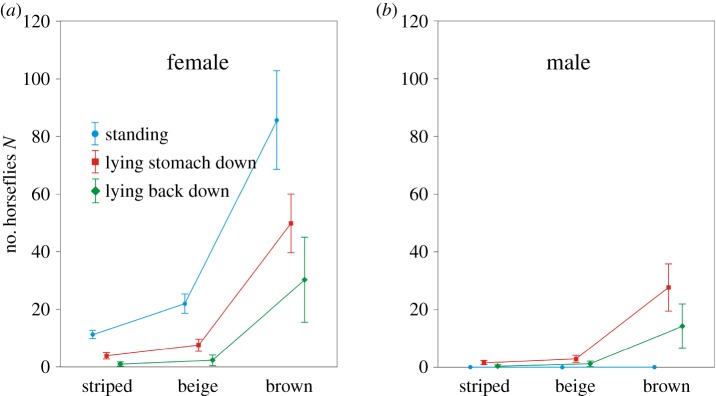
Number of trapped horseflies on different human models. Weighted means of the numbers of female and male horseflies trapped by the sticky white-striped brown, beige and brown human models (electronic supplementary material, table S1). Vertical I-shaped bars denote 0.95 confidence intervals.

Using factorial ANOVA, we found significant differences in the numbers of horseflies trapped (henceforth referred to as ‘catches’) considering the posture and colour of the human models as well as the sex of the horseflies. Combining these effects, the results were also significant (figure 5; electronic supplementary material, tables S1 and S2). When the sticky human models were in standing posture, they trapped only female horseflies (figure 5; electronic supplementary material, table S1). In the standing posture, the brown model was the most attractive, capturing 72% (*N* = 1200 females) of the total catch, the beige model caught 18.5% (308) and the white-striped brown model was the least attractive, capturing only 9.5% (158). The brown model was 1200/158 = 7.6 times more attractive to horseflies than the white-striped brown model (Tukey HSD post hoc test *p* < 0.0001, significant), the beige model attracted 308/158 = 1.9 times more horseflies than the white-striped brown model (*p* = 0.3039, non-significant) and the attractiveness of the brown model was 1200/308 = 3.9 times larger than that of the beige model (*p* < 0.0001, significant). The degree of freedom (d.f.) of the Tukey HSD post hoc tests was 150.

When the human models were lying on the ground (stomach or back down), they trapped both female and male horseflies. In the horizontal posture, the brown model was again the most attractive capturing 83.0–90.4% of the total catch. The least attractive was the white-striped brown model as it captured just 2.6–5.8% of the horseflies and the beige model was inbetween as it caught 7.0–11.2% of the horseflies (electronic supplementary material, table S1). The brown model was 543/38–312/9 = 14.3–34.7 times more attractive than the striped brown model (*p* < 0.0001, significant for both lying stomach down and back down; electronic supplementary material, tables S1 and S2). The beige model attracted 73/38–24/9 = 1.9–2.7 times more horseflies than the striped brown model (*p* = 0.999, not significant for both lying belly down and back down), and the attractiveness of the brown model was 543/73–312/24 = 7.4–13.0 times larger than that of the beige model (*p* < 0.0001, significant for both lying stomach down and back down, see figure 5 and electronic supplementary material, table S1). The female/male ratio of the trapped horseflies was 1.8–2.1 for the lying brown model, 2–2.7 for the lying beige model and 2.5–3.5 for the lying striped brown model.

Considering the total catches of horseflies that were trapped on the human models (pooled data of the standing and lying postures), the brown, beige and white-striped brown models trapped 77.1, 15.2 and 7.7% of horseflies with a female/male ratio of 6.0, 13.5 and 14.8, respectively (electronic supplementary material, table S1). Overall the brown model was 2055/205 = 10.0 times more attractive to horseflies than the striped brown model (*p* < 0.0001, significant). The beige model attracted 405/205 = 2.0 times more horseflies than the striped brown model (*p* = 0.1457, non-significant), while the attractiveness of the brown model was 2055/405 = 5.1 times larger than that of the beige model (*p* < 0.0001, significant).

We conclude that white-striped bodypaintings on brown skin have the advantageous effect of protecting against blood-sucking horseflies by making the skin surface less visually attractive to horseflies compared with homogeneous brown-skinned human bodies. The attractiveness to horseflies of a white-striped brown human body surface is 50% lower than that of a homogeneous beige body.

## 4. Discussion

There is good evidence that (i) horizontally polarizing water attracts male and female horseflies, (ii) strongly polarizing (dark) host animals (mainly ungulates) attract female horseflies, (iii) striped/spotted targets are unattractive to horseflies and (iv) striped bodypainting is common in many indigenous tribal communities. These factors lead to the following hypotheses that we test here: (1) Dark humans with striped bodypaintings may be less attractive to horseflies than homogeneous dark- or lighter-skinned humans. (2) The attractiveness of the human body to tabanids depends on its posture (standing versus lying). Hence, using standing and lying human models, the shape and posture of which are quite different from those of the host animals of horseflies, the novelty of our study relative to some previous findings (e.g. [26,27]) is that it corroborates these two hypotheses.

Bodypainting has been a widespread tradition of certain indigenous African, Australian, Papua New Guinean and North American tribes (figures 1 and 2). As a temporary mark, bodypaintings last only a few days. The colours and designs used in tribal bodypainting are chosen according to strict social and religious guidelines [17]. There are many body decoration motifs in Africa, Australia and elsewhere, but stripes are a common element. In this work, we present experimental evidence that striped bodypaintings confer protection against biting horseflies by making the body less visually attractive to horseflies.

The results of our field experiment support the theory that the use of striped bodypainting may be related to protection against dangerous parasitic insects. We found that striped bodypainting reduces the visual attractiveness of bodies to horseflies. However, we would like to emphasize that the primary reasons for the use of bodypaintings are social and cultural [1,3,4,19,20,39–42] and the protection these bodypaintings may confer against horseflies was unlikely to be a determining issue in their origin.

Bodypainting is normally a temporary part of social activities. If bodypainting was only intended to repel horseflies, people would be likely to wear the bodypaint permanently. Thus, we find it plausible that deterring horseflies is simply an advantageous byproduct of bodypainting. Women from the ethnic African shepherds of the Himba tribe (Namibia) have been reported to claim that a paste of butter, powdered ochre and herbs protect them from the sun, dry air and biting insects [43–45]. This suggests that both African and Aboriginal people may be familiar with the parasite-repelling feature of their bodypaintings.

Blood-sucking female horseflies are attracted to their hosts by multiple olfactory [28,46,47] and visual cues. The latter could be the shape, movement, brightness/darkness and colour of the host [48,49], as well as the linear polarization of host-reflected light [23,26,29,31,32,27]. It has previously been shown that striped or spotted targets are significantly less attractive to host-seeking female horseflies than homogeneous white or black targets [26,27], even when they emit typical host-born olfactory attractants, such as CO_2_ and ammonia which are known to attract horseflies [36]. In our present field experiment, we found that the brown human model was approximately 10 times more attractive to horseflies than the white-striped brown model. This corroborates our previous findings [26,27,36].

There are differences between humans and the human models used in our experiments. A range of factors could come into play, such as the nature of the base material, as well as the temperature and odour of the target. However, the physical characteristics (smoothness and stickiness) of the surface of our three human models were the same. Although the surface temperatures of our models were different in the sunshine (dark brown surfaces were warmer than white and light beige ones), this is unlikely to have affected our results as horseflies can detect temperature only after landing on a surface, rather than by remote sensing during flight [35,50].

The chemical nature of the white oil paint used in our experiment for white stripes on the dark brown human model was different from that used by African and Aboriginal people, who apply white clay, chalk, ash and various bright minerals dug out from a river-bank. However, only the intensity and polarization of reflected light is of importance in terms of the optical imitation of the white stripes. The white oil paint stripes and the white clay/chalk/ash/mineral stripes should have similarly high brightness (light intensity) and low degree of polarization as a trivial physical/optical consequence of the rule of Umow [51]. The white oil paint stripes on one of our two brown human models were completely dry and odourless (the diluent in the white paint had evaporated during the weeks preceding the field experiment). The painted plastic surfaces of our human models were also dry and odourless. Even if the white stripes or the plastic surface of the models emitted some minimal remnant odour, this could not influence their attractiveness to horseflies, because the whole surface of all three models was covered by the same insect-monitoring glue layer, that prevented odour evaporation from the surfaces. Hence, the significant difference between the models in their attractiveness to horseflies cannot be explained by different olfactory cues. Thus, the difference in composition between our white paint stripes and the white clay/chalk/ash/mineral stripes of bodypaintings cannot influence our results. The thin, odourless, transparent, colourless glue layer covering the human models did not significantly alter the brightness and degree of polarization of reflected light, which are the optical characteristics that determine the visual attractiveness of an object to horseflies. It has been shown that the thickness of stripes plays an important role in how attractive a surface is to horseflies: horseflies are less attracted to surfaces with thinner stripes compared to those with wider stripes ([26], reviewed by Caro [25]). In our experiment, we used stripes with a width of 4 cm interspersed by 4–5 cm on the white-striped brown human model. This stripe width, which represents the medium width of stripes used in human body paintings (figures 1 and 2), does not pose a problem for the interpretation of our results as thinner and more numerous stripes would have resulted in these models being even less attractive to horseflies.

Here we show that human traditional bodypaintings with their typical white-striped patterns on a brown body surface have the advantage of deterring blood-sucking horseflies as these patterns are unattractive to these parasitic insects. We found that these bodypaintings are less attractive to horseflies than a homogeneous dark brown or a homogeneous bright beige human model. This feature of bodypaintings is very useful, because on the one hand, host-seeking female horseflies can intolerably annoy people, and on the other hand, due to their blood-sucking habits they are vectors of the pathogens of several diseases and/or parasites (e.g. tularemia, anaplasmosis, hog cholera, equine infectious anaemia, filariasis, anthrax, Lyme disease) and thus are dangerous or even lethal to humans [52–55]. It is well known that horseflies prefer to attack dark mammals for a blood meal [23,26,27,36]. Consequently, humans with dark brown skin are likely to be particularly attractive to host-seeking horseflies. However, this attractiveness to horseflies can, as we have shown here, be reduced by white-striped bodypaintings. Interestingly, a similar effect of white stripes has been demonstrated in zebras [26,36], and therefore horseowners have started to protect their dark-coated horses against attacking horseflies by the use of zebra-striped horseclothes (http://www.equiporium.co.uk/bucas-buzz-off-full-neck-zebra-fly-rug.html, http://www.saddlekraft.com/horse-fly-rugs.html).

Since horseflies are currently abundant throughout the whole year in the tropical regions of Africa [7–10,22,56–63], Australia [11–13] and Papua New Guinea [14], bodypainting is likely to confer protection against horseflies all year-round in these regions. We do not state that the original purpose of bodypainting was to deter horseflies, but we have experimentally demonstrated that striped white bodypaintings reduce the risk of being attacked by blood-sucking horseflies, and consequently decrease the chance of being exposed to bites and diseases transmitted by these insects.

Our finding that white-striped bodypaintings protect dark-skinned humans against horseflies was expected, because a similar result has been obtained in earlier field experiments performed with striped horse models and horseflies [26,36]. However, the shape of a human in a standing posture is quite different from the shape of a quadrupedal animal. We studied whether human models in either a lying or standing posture were differentially attractive to horselflies, whereas earlier studies have only tested models of horses in a standing posture [26,36]. Horses and other ungulates as typical host animals attract only host-seeking female horseflies [23,26–29,31–34,36,46–49], while dark-coloured human models in a lying posture attract both water-seeking male and female horseflies. The visual attractiveness to horseflies of these two differently shaped targets is partly governed by their different polarotaxes [32]: (i) Water-seeking males and females are attracted by horizontally polarized light, because water surfaces usually reflect such light [35,37,38]. (ii) Host-seeking females are attracted by the high degree of polarization of light reflected by dark hosts, independently of the direction of polarization [30]. Recently, Horváth *et al*. [30] demonstrated that horseflies need polarization vision for host detection, because polarized light helps horseflies select sunlit dark host animals from the dark patches of the visual environment. However, the thorough studies of Caro *et al*. [64], Caro & Stankowich [65], Caro [25] and Melin *et al*. [66] showed that polarization of host-reflected light is not the only factor affecting horsefly behaviour during host detection.

Horseflies are associated with water, because their larvae develop in water or mud. Thus, they are abundant in areas where water bodies occur within several tens of kilometres—they can fly more than 100 km [50]. There is a positive correlation between the areas inhabited by African/Aboriginal people using bodypainting and the distribution of horseflies, because these insect parasites occur wherever there are water bodies (table 1). Although in the most arid parts of central Australia with Aboriginal communities using bodypainting horseflies are relatively rare [67], in several non-arid regions of Australia and Africa with people using bodypaintings horseflies are abundant. Striped patterns also defend against blood-sucking tsetse flies [24,68] occurring in many areas of Africa. These parasites (both the females and males of which are blood-sucking, while in horseflies only the females are haematophagous) are not associated with water, therefore they also occur in arid regions. Thus, white-striped bodypaintings are unattractive not only to horseflies, but also to tsetse flies, which is a further advantage in Africa.

## 5. Conclusion

In our field experiment, the model that was least attractive to horseflies was the white-striped dark model, followed by the beige model and then the dark human model. Male horseflies were only attracted to the dark human model in a lying posture. Thus, tabanid flies prefer homogeneous dark human bodies, while white-striped bodypaintings on dark-skinned humans deter these blood-sucking flies.

## Supplementary Material

Electronic Supporting Material

## Data accessibility

Our paper has the following electronic supplementary material: tables S1, S2 and figures S1, S2 and S3.
