# Supplementary material for: Striped bodypainting protects against horseflies
Source: R Soc Open Sci. 2019 Jan 16;6(1):181325. doi: 10.1098/rsos.181325 (PMC6366178; doi:10.1098/rsos.181325)
Supplement: Electronic Supporting Material [file rsos181325supp1.doc]

**Electronic Supplementary Material**

**for**

**Striped bodypainting protects against horseflies**

Gábor Horváth^1^,*, Ádám Pereszlényi, Susanne Åkesson and György Kriska

1: Environmental Optics Laboratory, Department of Biological Physics, ELTE Eötvös Loránd University, H-1117 Budapest, Pázmány sétány 1, Hungary

*: correspondig author, e-mail: gh@arago.elte.hu

This file contains the following: Supplementary Tables S1, S2

Supplementary Figures S1, S2, S3

**Supplementary Table S1**: Number of horseflies trapped by the three differently coloured sticky human models (white-striped brown, beige, brown) for their three different postures (standing, lying on ground with stomach down, lying on ground with back down) in our choice experiment. f: female, m: male, f/m: female/male ratio. The standing human models trapped only female horseflies.

| **posture of**  **human models** | **date (2015)** | **human models** | | |
| --- | --- | --- | --- | --- |
|  |  | **white-striped brown**  **(Fig. S3D)** | **beige**  **(Fig. S3E)** | **brown**  **(Fig. S3F)** |
| standing  (Fig. S3A) | 22-23 June | 9 | 20 | 92 |
|  | 24-25 June | 12 | 22 | 90 |
|  | 26-27 June | 10 | 22 | 99 |
|  | 28-29 June | 9 | 21 | 95 |
|  | 30 June - 1 July | 14 | 31 | 110 |
|  | 2-3 July | 12 | 22 | 116 |
|  | 4-5 July | 16 | 33 | 123 |
|  | 6-7 July | 9 | 21 | 98 |
|  | 8-9 July | 11 | 20 | 86 |
|  | 10-11 July | 15 | 25 | 100 |
|  | 12-13 July | 13 | 25 | 81 |
|  | 14-15 July | 11 | 22 | 57 |
|  | 16-17 July | 10 | 14 | 31 |
|  | 18-19 July | 7 | 10 | 22 |
|  | **sum** | **158 f = 9.5%** | **308 f = 18.5%** | **1200 f = 72.0%** |
| lying on ground,  stomach down  (Fig. S3B) | 20-21 July | 8 (3 m + 5 f) | 11 (4 m + 7 f) | 76 (25 m + 51 f) |
|  | 22-23 July | 5 (2 m + 3 f) | 15 (5 m + 10 f) | 80 (25 m + 55 f) |
|  | 24-25 July | 5 (1 m + 4 f) | 11 (3 m + 8 f) | 89 (33 m + 56 f) |
|  | 26-27 July | 7 (2 m + 5 f) | 13 (2 m + 11 f) | 79 (29 m + 50 f) |
|  | 28-29 July | 6 (1 m + 5 f) | 7 (2 m + 5 f) | 37 (11 m + 26 f) |
|  | 30-31 July | 4 (1 m + 3 f) | 10 (3 m + 7 f) | 84 (32 m + 52 f) |
|  | 1-2 August | 3 (1 m + 2 f) | 6 (1 m + 5 f) | 98 (39 m + 59 f) |
|  | **sum** | **38 (11 m + 27 f) =**  **5.8% (f/m = 2.5)** | **73 (20 m + 53 f) =**  **11.2% (f/m = 2.7)** | **543 (194 m + 349 f) =**  **83.0% (f/m = 1.8)** |
| lying on ground,  back down  (Fig. S3C) | 3-4 August | 2 (1 m + 1 f) | 3 (1 m + 2 f) | 51 (15 m + 36 f) |
|  | 5-6 August | 3 (1 m + 2 f) | 6 (2 m + 4 f) | 79 (29 m + 50 f) |
|  | 7-8 August | 2 (0 m + 2 f) | 9 (3 m + 6 f) | 70 (20 m + 50 f) |
|  | 9-10 August | 0 | 3 (1 m + 2 f) | 43 (14 m + 29 f) |
|  | 11-12 August | 1 (0 m + 1 f) | 2 (1 m + 1 f) | 33 (10 m + 23 f) |
|  | 13-14 August | 1 (0 m + 1 f) | 1 (0 m + 1 f) | 20 (7 m + 13 f) |
|  | 15-16 August | 0 | 0 | 16 (5 m + 11 f) |
|  | **sum** | **9 (2 m + 7 f) =**  **2.6% (f/m = 3.5)** | **24 (8 m + 16 f) =**  **7.0% (f/m = 2)** | **312 (100 m + 212 f) =**  **90.4% (f/m = 2.1)** |
| **total** | | **205 (13 m + 192 f) =**  **7.7% (f/m = 14.8)** | **405 (28 m + 377 f) =**  **15.2% (f/m = 13.5)** | **2055 (294 m + 1761 f) =**  **77.1% (f/m = 6.0)** |

**Supplementary Table S2**: Univariate tests of significance for the data of Supplementary Table S1. SS: sum of squares, DF: degree of freedom, MS: mean of squares, F: Fisher criteria, p: degree of significance. Intercept means data without factor effects. All p-values are significant.

| **effect** | **SS** | **DF** | **MS** | **F** | **p <** |
| --- | --- | --- | --- | --- | --- |
| **intercept** | 31964.04 | 1 | 31964.04 | 314.9432 | 0.0001 |
| **posture** | 3784.83 | 2 | 1892.41 | 18.6460 | 0.0001 |
| **colour** | 30776.02 | 2 | 15388.01 | 151.6188 | 0.0001 |
| **sex** | 12859.47 | 1 | 12859.47 | 126.7050 | 0.0001 |
| **posture and colour** | 1377.19 | 4 | 344.30 | 3.3924 | 0.0109 |
| **posture and sex** | 10714.59 | 2 | 5357.29 | 52.7857 | 0.0001 |
| **colour and sex** | 9989.50 | 2 | 4994.75 | 49.2135 | 0.0001 |
| **posture and colour and sex** | 6530.71 | 4 | 1632.68 | 16.0869 | 0.0001 |
| **error** | 15223.71 | 150 | 101.49 |  |  |


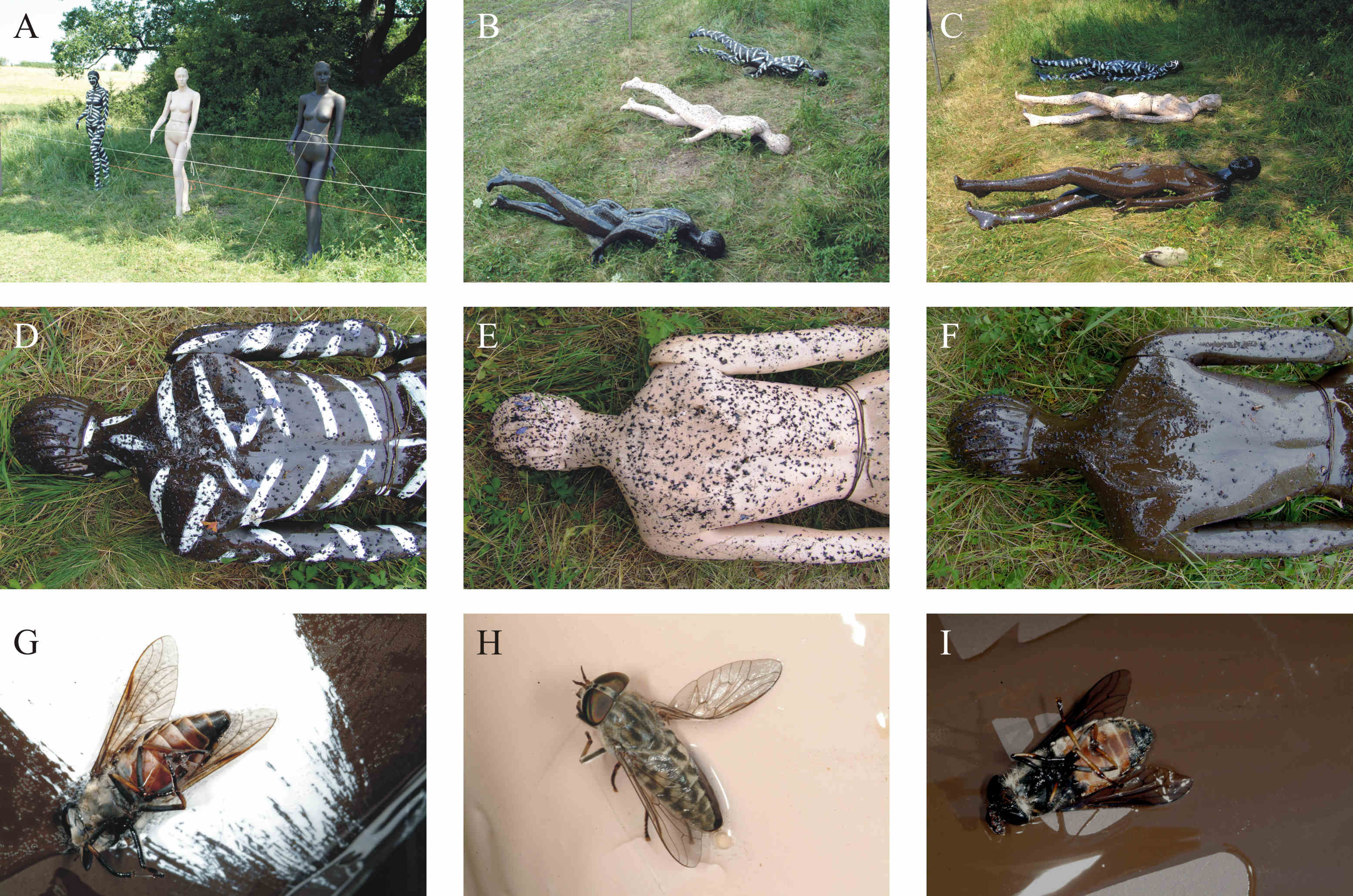


**Supplementary Figure S1**: **Experimental setup.** Photographs of the sticky white-striped brown (D), beige (E), and brown (F) human models used in the choice experiment when they were standing (A), lying on the ground with stomach down (B), and lying on the ground with back down (C). (D-F) The sticky body surface of the models trapped numerous insects, especially flies, many of which were horseflies. (G-I) Photos of horseflies trapped by the white-striped brown (G), beige (H), and brown (I) human models.


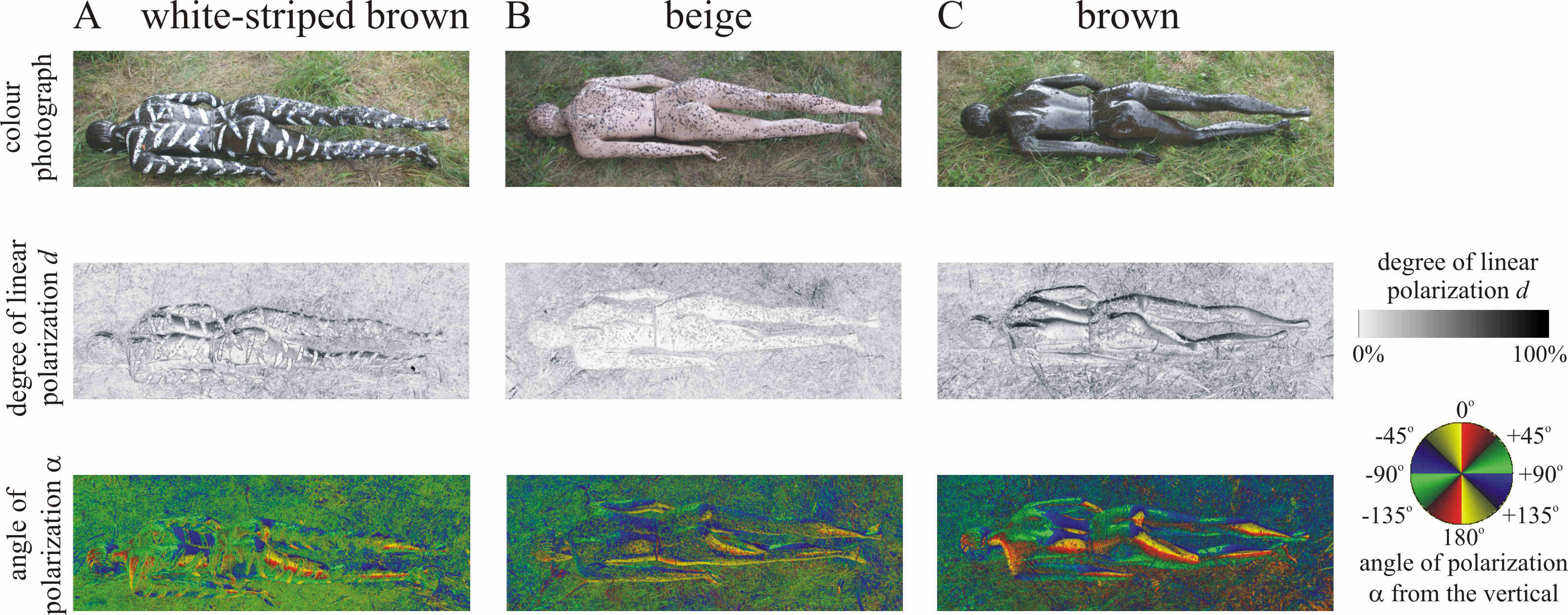


**Supplementary Figure S2**: As Fig. 3 measured perpendicularly to the long axis of the human models from their left hand side.


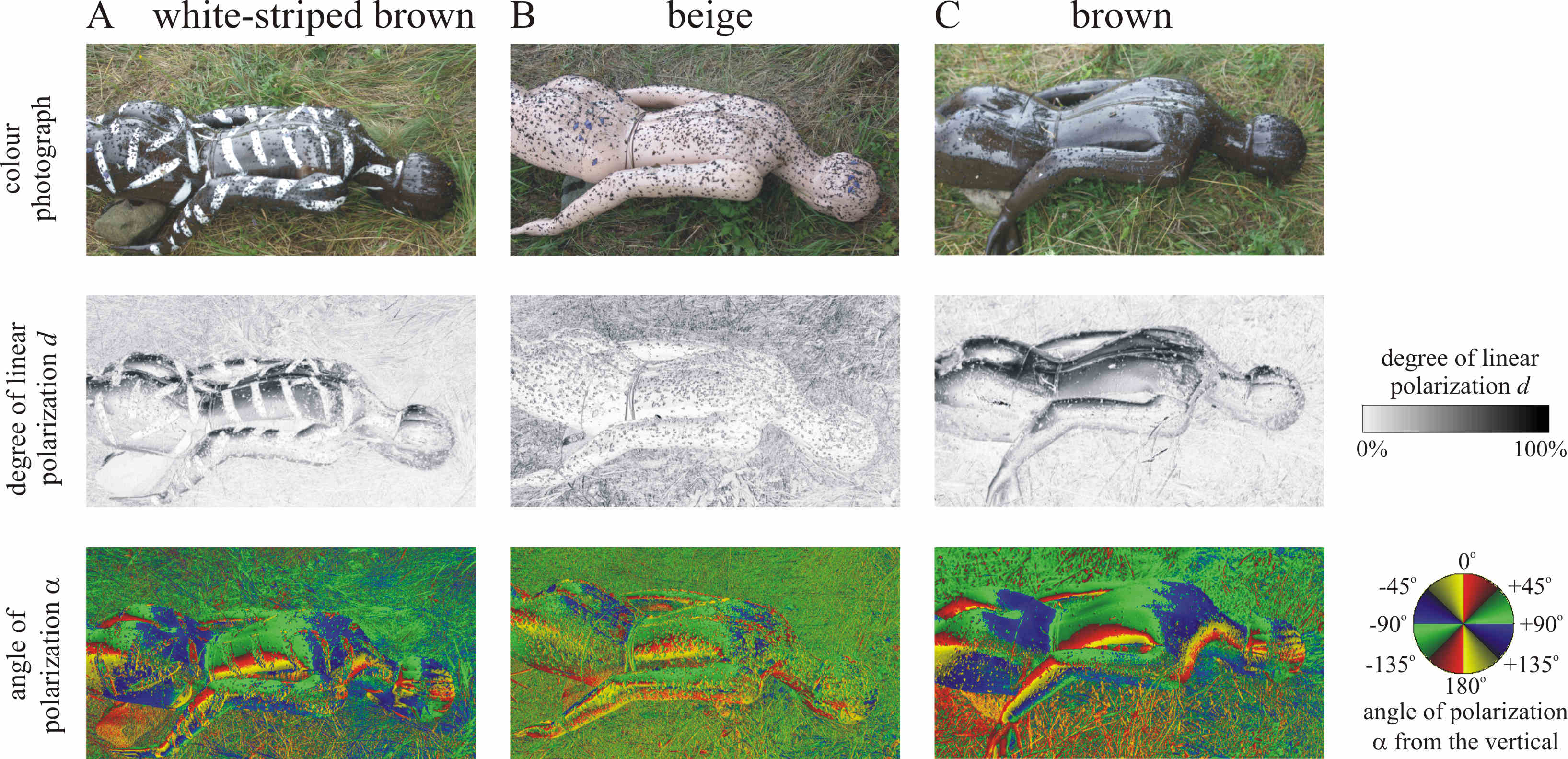


**Supplementary Figure S3**: As Fig. 3 measured from the right hand side of the human models.
